# Participation behaviour of different migrant groups in breast cancer screening – palpation of the breast and mammography. Results from the German national cohort (NAKO)

**DOI:** 10.1016/j.jmh.2026.100397

**Published:** 2026-01-13

**Authors:** Heiko Becher, Nadia Obi, Tilman Brand, Hermann Brenner, Laura Buschmann, Renée T. Fortner, Karin Halina Greiser, Volker Harth, Wolfgang Hoffmann, André Karch, Thomas Keil, Alexander Kluttig, Lilian Krist, Michael Leitzmann, Andy Maun, Rafael Mikolajczyk, Katharina Nimptsch, Tobias Pischon, Sabine Schipf, Börge Schmidt, Ulla T. Schultheiss, Matthias Schulze, Hajo Zeeb, Christian Wiessner

**Affiliations:** a Heidelberg Institute of Global Health, University Hospital Heidelberg, Heidelberg, Germany; b Institute for Medical Biometry and Epidemiology, Hamburg University Medical Center, Hamburg, Germany; c Institute for Occupational and Maritime Medicine (ZfAM), University Medical Center, Hamburg, Germany; d Leibniz-Institute for Prevention Research and Epidemiology-BIPS, Bremen, Germany; e Division of Clinical Epidemiology and Aging Research, German Cancer Research Center (DKFZ), Heidelberg, Germany; f Universität Münster, Institut für Epidemiologie und Sozialmedizin, Münster, Germany; g Division of Cancer Epidemiology, German Cancer Research Center, Heidelberg, Germany; h Department of Research, Cancer Registry of Norway, Norwegian Institute of Public Health, Oslo, Norway; i Institut für Community Medicine, Universitätsmedizin Greifswald, Greifswald, Germany; j Institute of Social Medicine, Epidemiology and Health Economics, Charité - Universitätsmeditzin Berlin, Berlin, Germany; k Institute of Clinical Epidemiology and Biometry, University of Würzburg, Würzburg, Germany; l State Institute of Health I, Bavarian Health and Food Safety Authority, Erlangen, Germany; m Institute for Medical Epidemiology, Biometrics, and Informatics, Interdisciplinary Center for Health Sciences, Medical Faculty of the Martin Luther University Halle-Wittenberg, Halle (Saale), Germany; n Department of Epidemiology and Preventive Medicine, University of Regensburg, Regensburg, Germany; o Institute Institute of General Practice / Family Medicine, Faculty of Medicine and Medical Center, University of Freiburg, Germany; p Max-Delbrueck-Center for Molecular Medicine in the Helmholtz Association (MDC), Molecular Epidemiology Research Group, Berlin, Germany; q Max-Delbrueck-Center for Molecular Medicine in the Helmholtz Association (MDC), Biobank Technology Platform, Berlin, Germany; r Charité - Universitätsmedizin Berlin, corporate member of Freie Universität Berlin and Humboldt-Universität zu Berlin, Germany; s Institute for Medical Informatics, Biometry and Epidemiology, University Hospital of Essen, University of Duisburg-Essen, Germany; t Institute of Genetic Epidemiology, Faculty of Medicine and Medical Center - University of Freiburg, Freiburg, Germany; u Department of Molecular Epidemiology, German Institute of Human Nutrition Potsdam-Rehbruecke, Nuthetal, Germany; v Institute of Nutritional Science, University of Potsdam, Nuthetal, Germany; w Health Sciences Bremen, University of Bremen, Bremen, Germany

**Keywords:** Migrants, Breast cancer, Mammography screening, Breast palpation, Participation behaviour

## Abstract

**Background:**

For early detection of breast cancer, clinical palpation of the breast is offered yearly to all women aged 30 and older, and the German Mammography Screening Programme (MSP) offers biennial mammograms to all women aged 50 to 75 years. We investigated the utilization of both screening methods across various migrant groups in Germany, as well as the effect of German language proficiency.

**Methods:**

Cross-sectional data on participation frequencies from the baseline examination (2014 to 2019) of more than 100,000 women of the German National Cohort study (NAKO) were analysed by migrant status. Adjusted logistic regression analyses were conducted for palpation and MSP to compare screening uptake among six migrant groups, and non-migrant population.

**Results:**

Palpation of the breast was less frequently utilized in all migrant groups with odds ratios ranging from 0.5 (95% CI 0.4–0.6) for Turkish women to 0.9 for women from western countries (95% CI 0.7–1.1) compared to autochthone Germans. Lower German language proficiency further decreases its use. In contrast, odds ratios for MSP participation did not differ substantially compared to Germans ranging from 0.8 to 1.2. German language proficiency had little effect on MSP participation.

**Discussion:**

In contrast to earlier studies, our findings suggest that MSP participation and motivation does not significantly differ by migration status or language skills. This may indicate that information on MSP is broadly accessible through established invitation procedures in Germany. However, lower uptake of breast palpation by a physician in some migrant populations highlights potential gaps in broader preventive care engagement.

## Highlights

• breast cancer screening programs in Germany covered by health insurance include palpation of the breast by a physician (from age 30) and mammography (age 50 to 75).
• Palpation of the breast was less frequently utilized in all migrant groups with odds ratios ranging from 0.5 (95 % CI 0.4–0.6) for Turkish women to 0.9 for women from western countries (95 % CI 0.7–1.1) compared to autochthone Germans.
• Lower German language proficiency further decreases its use.
• Mammography participation did not differ substantially compared to Germans with odds ratios ranging from 0.8 to 1.2.
• German language proficiency had little effect on mammography participation.

## Introduction

Breast cancer is the most common female cancer in Germany with an estimated number of 70,550 incident cases and 18,425 deaths in the year 2020, which corresponds to a standardised incidence and mortality rate of 112.7 and 21.8 per 100,000, respectively, standardised to the European Standard Population (Koch-Institut, 2023). Since 2009, all women aged between 50 and 69 years with a permanent residence in Germany are invited to participate in the biennial Mammography Screening Programme (MSP) (Kooperationsgemeinschaft Mammographie 2024). Similar MSP programmes exist in many countries worldwide, albeit with varying guidelines and participation rates as recently reviewed by Ren et al. (Ren et al., 2022). The aim of such programmes is to reduce breast cancer mortality by earlier detection (Autier et al., 2024, Dibden et al., 2020). In Germany, the MSP participation rate in 2022, calculated as the proportion of invited women who took part in the MSP in that year, was 50.4 % (Kooperationsgemeinschaft Mammographie 2024). Clinical breast palpation is not part of the MSP and consequently it is not performed in the screening units. However, it is commonly conducted in routine care, primarily by gynaecologists. In Germany, all women aged 30 and over are entitled to this early detection examination once a year by gynaecologists, which is covered by statutory health insurances.

Previous national and international studies have shown that the participation behaviour of eligible women is influenced by demographic, socio-economic, educational, and behavioural factors (Mottram et al., 2021). Buschmann et al. (Buschmann et al., 2025) used data from the German National Cohort (NAKO) to investigate in detail factors that may explain the MSP participation behaviour in Germany (Peters et al., 2022) and compared it with other studies. They found family history, participation in other screening programmes, general health awareness and a high social network index (Lubben and Matz-Costa, 2021) to be associated with a higher participation in MSP. With regard to migrant research, several studies have consistently shown disparities in mammography uptake between migrant and non-migrant women in various countries including Austria (Wahidie et al., 2024), Germany (Kaucher et al., 2020, Lemke et al., 2015, Berens et al., 2015), Denmark (Kristiansen et al., 2012), Australia (Weber et al., 2009), New Zealand (Zhang et al., 2014), Turkey (Tuzcu and Bahar, 2015), and the United States (Poonawalla et al., 2014). Analyses of large-scale survey data, such as the Austrian Health Interview Survey (Wahidie et al., 2024), revealed significantly lower screening participation among migrant women compared to non-migrants, even after adjusting for sociodemographic variables. In Germany, resettlers from the former Soviet Union demonstrated higher mammography screening rates compared to the general population; however, this study was small and included 69 resettlers only (Kaucher et al., 2020). Quantitative studies from Australia, including data from the 45 and Up Study (Weber et al., 2009), further confirmed that migrant women were less likely to participate in MSP, with participation rates varying by region of origin and length of stay. Qualitative studies from Denmark (Kristiansen et al., 2012), Turkey (Tuzcu and Bahar, 2015), and New Zealand (Zhang et al., 2014) provided additional insights into cultural and transnational influences on screening behaviours but were limited by smaller sample sizes (Lubben and Matz-Costa, 2021). Small-area spatio-temporal analyses in Germany indicated regional and socioeconomic disparities in mammography programme participation (Lemke et al., 2015).

Recent data indicate that a substantial proportion (28.7 %) of Germany’s population has a migration background (Federal Ministry Of The Interior, Federal Office For Migration And Refugees 2024). This figure does not include refugees without a legal residency status. Among those with a migration background, 63.8 % are first-generation migrants, while 35.2 % were born in Germany to at least one migrant parent. Migrants are highly heterogeneous with regard to nationalities, time since arrival in the host country, reasons for migration, language proficiency and regions of origin (Kajikhina, 2023). Therefore, using “migration background” as a binary variable in public health research is insufficient. In Germany, the largest subgroups are individuals with Turkish background, and those who migrated to Germany as descendants from Germans who migrated to Russia in the 17th to 19th century (so-called “resettlers”, german: “(Spät-)Aussiedler”). Other significant groups include individuals from other Eastern European countries, Asia, Western countries, and the Middle East.

Collectively, these findings underscore the importance of systematically investigating the two screening methods, palpation of the breast and mammography, among different migrant groups in large, population-based cohorts such as the NAKO. This study aims to analyse participation behaviour in breast cancer screening among migrant groups in Germany in detail and to assess whether targeted and specific measures to enhance screening uptake are warranted.

## Data and methods

### Data

We used data from the NAKO which aims to investigate the causes of major common diseases, identify risk factors and improve the early detection and prevention of diseases (Peters et al., 2022). Between March 2014 and September 2019, 204,733 individuals aged 20 to 69 years were randomly selected from residents' registration offices with an average response rate of 16 % (Rach et al., 2025). The baseline examination was conducted in 18 study centres distributed across Germany and included a detailed standardised interview and self-administered questionnaires as well as medical examinations and the collection of biomaterials. Regarding breast cancer screenings, the following question was asked to ascertain information on several cancer screening programmes: “In the last 5 years, have you had one or more of the following examinations carried out once or more than once? (i) Palpation examination of the breast by a physician, (ii) X-ray examination of the breast (‘mammography’, early detection of breast cancer”).

For the MSP analysis, we first selected all women in the age range which entitled them to participate in the screening programme (50–74 years). This resulted in 55,588 women. After excluding 5838 women who had missing information on the MSP and an additional 32 on migration status, the final analytic sample consisted of 49,718 women.

**Fig. 1:**
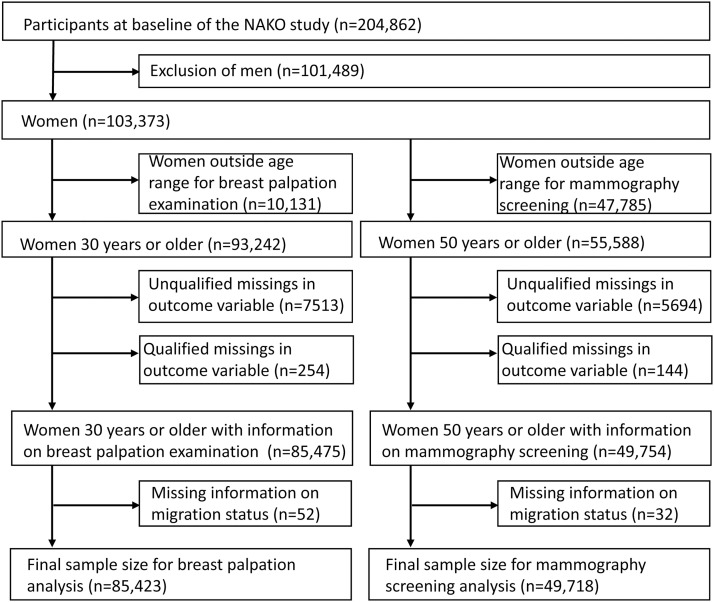
▒.

For the palpation analysis, we considered an extended age range of 30–74 years. Of 93,242 eligible women, 7767 had missing information on palpation and 52 lacked migration data. Thus, the final sample for this analysis included 85,423 women, which included the sample used for the MSP analysis. See the flowchart which illustrates the selection of the study population (Fig. 1).

Migration background was assigned according to the definition of the Federal Statistical Office of Germany (Federal Ministry Of The Interior, Federal Office For Migration And Refugees 2024). This classification considers the nationality and country of birth of the participant and both parents. First generation migrants were categorised as those born without a German nationality and with a personal migration experience to Germany, while second generation migrants were assigned a migration status if at least one parent was born without a German nationality and had a migration experience. First generation migrants were further grouped into different regions according to their country of birth, based on the definition of the United Nations (United Nations 2019), and were categorised into the following subgroups (Wiessner et al., 2024):

• Western migrants (Western Europe, Northern Europe, Southern Europe, North America, Australia)
• Migrants from the former Soviet Union with German ancestors (resettlers)
• Eastern Europe migrants (except resettlers from the former Soviet Union and Turkish migrants)
• Turkish migrants
• Other migrants (Latin America, Africa, Asia)

Second generation migrants were not further subdivided since numbers became too small for such detailed analysis. A detailed description of the countries of origin of the NAKO cohort members is given in Wiessner et al. (Wiessner et al., 2020).

Mother tongues were assessed by own classification. Additionally, participants were asked about their German language skills ranging from very good to very bad. Individuals with German as their mother tongue were defined as having very good language skills. We classified women into four groups (mother tongue / very good, good, medium, low). It is important to note that sufficient language skills were listed as a prerequisite for participation in the NAKO since only in few cases interpreters were available for support. For this reason, only few people with very little knowledge of German are included in the NAKO and no firm conclusions can be drawn about this group of women.

### Statistical methods

We present descriptive analyses for all considered variables in tables or figures as appropriate. To assess the association between migrant status and language proficiency with screening participation within the last five years, we performed multivariable logistic regression analyses with migrant group and language proficiency simultaneously. MSP and palpation were analysed in separate models comparing at least one use of the respective screening programme vs. no use in the past 5 years. As adjustment variables we considered age (continuously), sociodemographic variables (education) and study center. We checked for possible clustering in the data by estimating robust (sandwich) standard errors accounting for clustering by study center. Education was categorised into low (International Standard Classification of Education (ISCED97)-level 1/2), medium (ISCED97-level 3/4), and high (ISCED97-level 5/6) (UNESCO 1997).

We used the software SAS 9.4 (SAS Institute Inc, 2023) for all analyses.

## Results

Table 1 presents the sociodemographic variables of the study population for (a) analysis of palpation of the breast (age range 30–74) and (b) MSP (age range 50–74) giving the mean (standard deviation) or the frequency (percentages) as appropriate. The distribution of age and education is relatively similar across all population groups - except for women with a Turkish background, who tend to be younger and, on average, have lower levels of educational level.

**Table 1:** Distribution of sociodemographic variables by migrant group, separately for MSP and palpation.

	Study sample for palpation of the breast analysis (age range 30–74)							
	No migration background	Second generation migrants	Eastern Europe migrants	Resettlers from former SU	Western migrants	Turkish migrants	Other^1^	total
N	72,174	4144	3289	1467	2005	826	1518	85,423
Age (mean, sd)	52.4 (10.2)	51.4 (10.5)	50.7 (10.9)	47.8 (10.4)	52.2 (10.7)	45.4 (8.3)	47.9 (9.8)	
Education Low	1515 (2.3 %)	115 (3.0 %)	112 (3.7 %)	49 (3.7 %)	149 (8.2 %)	148 (21.1 %)	116 (8.5 %)	2204 (2.8 %)
Medium	31,458 (46,8 %)	1685 (43.9 %)	1223 (40.7 %)	532 (39.7 %)	612 (33.8 %)	328 (46.9 %)	416 (30.3 %)	36,254 (45.7 %)
High	34,255 (51.0 %)	2038 (53.1 %)	1670 (55.6 %)	758 (56.6 %)	1050 (58.0 %)	224 (32.0 %)	840 (61.2 %)	40,835 (51.5 %)
Missing	4946	306	284	128	194	126	146	6130

	Study sample for MSP analysis (age range 50–74)							
	No migration background	Second generation migrants	Eastern Europe migrants	Resettlers from former SU	Western migrants	Turkish migrants	Other^1^	total
N	43,234	2264	1657	631	1129	215	588	49,718
Age (mean, sd)	59.2 (6.0)	59.3 (6.3)	59.9 (6.4)	58.1 (5.5)	60.0 (6.4)	56.1 (6.0)	58.0 (5.6)	
Education Low	1210 (3.0 %)	83 (4.0 %)	61 (4.1 %)	19 (3.4 %)	111 (11.1 %)	50 (31.0 %)	38 (7.4 %)	1572 (3.4 %)
Medium	19,668 (49.0 %)	987 (47.4 %)	664 (44.2 %)	208 (36.8 %)	395 (39.5 %)	62 (38.5 %)	187 (36.5 %)	22,171 (48.3 %)
High	19,224 (47.9 %)	1014 (48.7 %)	777 (51.7 %)	339 (59.9 %)	494 (49.4 %)	49 (30.4 %)	288 (56.1 %)	22,185 (48.3 %)
Missing	3132	180	155	65	129	54	75	3790

SU – Soviet Union.

**MSP -** Mammography Screening Programme; sd – standard deviation; education - according to ISCED-97.

1 Migrants from Asia, Africa and Latin America.

Table 2 displays German language proficiency among individuals aged 30 and older, and 50 and older, stratified by migration background and specific migrant groups. As expected, individuals without a migration background report German as their mother tongue or rate their proficiency as very good (100 %). Among migrant groups, language proficiency varies considerably. In the 30+ age group, second-generation migrants (96.4 %) and migrants from the "other" category (mostly from Asia, Africa, and Latin America) show the highest proportions of very good or native-level German according to their self-judgement. In contrast, fewer migrants from Turkey (32.4 %) and the former Soviet Union (34.4 %) report very good proficiency, with a noticeable share having only medium or low German language skills. The trend continues among those aged 50 and older: people without a migration background and second generation migrants (96.5 %) show strong German skills. However, older migrants from Turkey and Eastern Europe are more likely to report only moderate or poor language skills, reflecting generational differences in language integration.

**Table 2:** Language skills by migrant group and by age ranges.

		Migration group							
	German N language % -level	No migration	Second generation migrants	Eastern Europe migrants	Resettlers from former SU	Western migrants	Turkish migrants	other	total
Age group 30–74 years	German mother tongue or very good	72,174 100.00	3978 96.46	1200 36.59	501 34.36	608 30.42	267 32.40	194 12.82	78,922
	Good	0 0.00	141 3.42	1642 50.06	681 46.71	1157 57.88	380 46.12	878 58.03	4879
	Medium	0 0.00	3 0.07	388 11.83	252 17.28	213 10.66	139 16.87	370 24.45	1365
	Low	0 0.00	2 0.05	50 1.52	24 1.65	21 1.05	38 4.61	71 4.69	206
	Total	72,174	4124	3280	1458	1999	824	1513	85,372
	Missing: 51								
Age group 50 – 74 years	German mother tongue or very good	43,234 100.00	2224 98.84	780 47.19	293 46.81	385 34.22	37 17.21	77 13.16	47,030
	Good	0 0.00	25 1.11	633 38.29	196 31.31	614 54.58	98 45.58	319 54.53	1885
	Medium	0 0.00	0 0.00	221 13.37	124 19.81	114 10.13	62 28.84	163 27.86	684
	Low	0 0.00	1 0.04	19 1.15	13 2.08	12 1.07	18 8.37	26 4.44	89
	Total	43,234	2250	1653	626	1125	215	585	49,688
	Missing: 30 other: migrants from Asia, Africa and Latin America; SU – Soviet Union								

Table 3, upper half, presents the frequencies and percentages of palpation of the breast by migrant group. The highest values were observed among individuals without migration background and second generation migrants, each with over 90 %. In contrast, the lowest proportions are found among women born in Turkey (82 %) and from other regions of the world (Asia, Africa and Latin America) (83 %). A somewhat different picture emerges for MSP as shown in Table 3, lower half. Here, the participation was relatively similar across most groups, with the exception of women from Turkey, who again showed the lowest proportion with 79.1 % compared to 85.5 % and 87.6 % in women without migration background and resettlers from the former Soviet Union.

**Table 3:** Frequency and proportion of palpation of the breast by a physician and MSP examination of the breast within the last 5 years by migrant group.

Palpation of the breast by a physician within the last 5 years (age group 30 and above)												
	Migration group											
N %	No migration background		Second generation migrants		Eastern Europe migrants		Resettlers from former SU	Western migrants	Turkish migrants	other	Total	
No	5,118 7.09		337 8.13		369 11.22		16,2 11.04	215 10.72	149 18.04	255 16.80	6,605 7.73	
Yes	67,056 92.91		3,807 91.87		2,920 88.78		1,305 88.96	1,790 89.28	677 81.96	1,263 83.20	78,818 92.27	
Total	72,174		4,144		3,289		1,467	2,005	826	1,518	85,423	
Missing: 7,738												
MSP examination of the breast within the last 5 years (age group 50 and above)												
No		6,266 14.49		369 16.30		253 15.27	78 12.36	167 14.79	45 20.93	89 15.14		7,267 14.62
Yes		36,968 85.51		1,895 83.70		1,404 84.73	553 87.64	962 85.21	170 79.07	499 84.86		42,451 85.38
Total		43,234		2264		1657	631	1129	215	588		49,718
Missing: 5870 other: migrants from Asia, Africa and Latin America												

MSP – mammography screening programme; SU – Soviet Union.

The results of the logistic regression analysis to assess the association for both palpation and MSP as dependent variables with migrant group and language proficiency as independent variables, and with adjustment for age, educational level, and study center are given in Table 4. For breast palpation, the results show that it is used more frequently in women without migration background, with the odds ratio estimates (OR) for migrants from Turkey, and for the mixed group of women from other countries (Asia, Africa and Latin America) being lowest (OR 0.50, 95 % confidence interval (CI) 0.40–0.64 and 0.54, 95 % CI 0.44–0.67, respectively). The language proficiency is closely associated with participation. Women with low proficiency have an OR of 0.23 (95 % CI 0.16–0.33) compared to women with high proficiency. Generally, with decreasing language skills the uptake of palpation screening decreases.

**Table 4:** The relation of migration and language proficiency with breast cancer screening programmes. Results of the logistic regression analysis.

		Palpation (*N* = 79,249)			MSP participation (*N* = 45,901)		
Variable	category	OR*	95 % CI		OR*	95 % CI	
Migration	No migration background	1.0			1.0		
	Second generation migrants	0.86	0.76 - 0.97		0.88	0.78 - 1.00	
	Resettler from former Soviet Union	0.75	0.61 - 0.93		1.25	0.95 - 1.65	
	Eastern Europe migrants	0.77	0.65 - 0.90		0.94	0.79 - 1.13	
	Turkish migrants	0.50	0.40 - 0.64		0.82	0.54 - 1.24	
	Western migrants	0.91	0.75 - 1.10		1.03	0.82 - 1.29	
	Asia, Africa and Latin America	0.54	0.44 - 0.67		1.09	0.81 - 1.47	
Language	German mother tongue /very good	1.0			1.0		
level	Good	0.83	0.70 - 0.97		1.00	0.81 - 1.23	
	Medium	0.43	0.36 - 0.53		0.93	0.71 - 1.23	
	Low	0.23	0.16 - 0.33		0.73	0.39 - 1.39	
Age	(per 10 years)	0.81	0.78 - 0.83		1.67	1.61 - 1.76	
Education	High	1.0			1.0		
	Medium	0.82	0.78 - 0.87		1.24	1.18 - 1.31	
	Low	0.45	0.40 - 0.51		0.95	0.82 - 1.09	
Goodness of fit statistics							
Deviance	Null Model		42,768.6			37,934.0	
	Full Model		41,855.0			37,286.9	
	LR-Test (DF, p-value)		913.6 (29, <0.0001)			647.2 (29,<0.0001)	

ISCED-97-Level: International Standard Classification of Education 97.

MSP – mammography screening programme.

LR – Likelihood ratio.

⁎ - adjusted for age, educational level and study center.

For MSP, the picture is different. Generally, the associations between participation and migrant status and language skills are small. Migrants from Turkey and second-generation migrants show the lowest OR with 0.82 (95 % CI 0.54–1.24) and 0.88 (95 % CI 0.78–1.00), respectively. For resettlers from the former Soviet Union, the OR is 1.25 (95 % CI 0.95–1.57). For the other migrant groups, the OR is close to 1, indicating no substantial differences compared to women without a migration background.

For both breast palpation and MSP, the results remained almost identical when allowing for clustering.

The global wald tests for the migration variable were significant for the palpation and not significant for the MSP model (Palpation: Chi-Square= 59.06, df=6, *p* < 0.001; MSP: Chi-Square= 9.35, df=6, *p* = 0.16).

## Discussion

Baseline data from the NAKO were used to investigate the participation behaviour of various female migrant groups in both the breast palpation examination and the MSP. Overall, the participation was comparable to earlier studies, with 92.3 % and 85.4 % of all women reporting at least one palpation or MSP examination within the past five years (Peters et al., 2022, Federal Ministry Of The Interior, Federal Office For Migration And Refugees 2024).

While the breast palpation procedure was used much less frequently in all migrant groups considered, the MSP participation rates were relatively consistent between migrant groups and the non-migrant population after adjusting for educational level. A possible explanation is a stronger reluctance in migrants to have the breast manually examined by a male gynaecologist while MSP does not require this. An information on the sex of the gynaecologist was unfortunately not available.

Our analysis suggests that MSP participation is not strongly influenced by German language proficiency. This finding contrasts with previous research from Germany and other countries, and is therefore both unexpected and encouraging. This suggests that the language barrier may not pose a major obstacle to participation in the organised breast cancer screening programme. However, this interpretation should be approached with caution for several reasons discussed below. In Buschmann et al. (Buschmann et al., 2025), family history, participation in other screening programs, general health awareness and a high social network index (Lubben and Matz-Costa, 2021) were associated with a higher participation in the MSP. While family history may be little confounded with migrant status, the social network index is likely to be higher in the autochthonous German population. The general health awareness, including participation in other screening programs, is likely to be correlated with participation in the MSP.

The main purpose of our study was to investigate the role of migration status in an explanatory way. In our analysis we considered those covariables only which are possible confounder. These were age and education since they were likely to be correlated with both participation of the screening procedures and with migration status. Other variable, as considered in Buschmann et al. (Buschmann et al., 2025), are not included here. Therefore, the models are not suitable to predict participation.

A discussion of risks and benefits of breast cancer screening is not a topic of this paper and we only refer to the review by Dibden et al. (Dibden et al., 2020). The invitation letter for MSP is provided (in German) at https://www.iqwig.de/download/p14–02_rapid-report_einladungsschreiben-und-merkblatt-zum-mammographie-screening.pdf and gives relevant information on the issue.

This study has several strengths and limitations. The NAKO is a very large cohort, and thus contains relatively large numbers from distinct migrant groups allowing a more refined analysis compared to studies that only employ a binary distinction between migrants and non-migrants. Additionally, assessment in the NAKO is highly standardised (Schipf et al., 2020) and all data used in our analyses underwent a thorough quality check.

However, certain limitations must be acknowledged. Migrants in Germany are very heterogeneous, and therefore the broad categories adopted in our analysis may not fully reflect all differences. The overall participation rate in the NAKO study was low, and previous analyses suggest a strong “healthy volunteer” effect (Rach et al., 2025). It is therefore likely that NAKO participants are more health conscious compared to the general population. This could imply that participants generally take part more often in MSP. This bias is likely present in both migrant and non-migrant groups but there may be uneven selection bias. In addition, because sufficient German language skills were a prerequisite for participation, the study may have disproportionately included more educated migrants and those with longer residence durations in Germany. This selection bias may attenuate true differences and bias results towards the null. While this limitation may underestimate differences in clinical palpation use among our participants, it is less clear whether it substantially affects MSP participation estimates, especially given the weak association between language proficiency and MSP uptake. An additional point is the wording of the questionnaire to assess MSP participation. Since it was asked for “x-ray examination of the breast”, this could also have occurred after the women reported any symptoms and not following an invitation to undergo screening. We think, however, that this holds for a small proportion only and does not appreciably alter the results.

In conclusion, MSP participation rates do not differ considerably between women with and without a migration background in Germany. This may suggest that the information on this screening programme and the motivation to participate are effectively and evenly reaching diverse segments of the population. In contrast, the lower uptake of breast palpation by a physician among migrant women highlights disparities in access or utilisation of more informal or opportunistic screening measures. Although palpation is of limited diagnostic value due to its low sensitivity and specificity, it remains a common screening method for younger women and non-participants of MSP. Therefore, efforts should continue to improve overall MSP participation while also addressing gaps in broader preventive care for migrant populations.

## Ethical statement

The German National Cohort (NAKO) study is performed with the approval of the relevant ethics committees, and is in accordance with national law and with the Declaration of Helsinki of 1975 (in the current, revised version).

## Funding

This project was conducted with data (Application No. NAKO-191) from the German National Cohort (NAKO) (www.nako.de). The NAKO is funded by the Federal Ministry of Education and Research (BMBF) [project funding reference numbers: 01ER1301A/B/C, 01ER1511D, 01ER1801A/B/C/D and 01ER2301A/B/C], federal states of Germany and the Helmholtz Association, the participating universities and the institutes of the Leibniz Association.

## CRediT authorship contribution statement

**Heiko Becher:** Writing – original draft, Project administration, Methodology, Funding acquisition, Formal analysis, Conceptualization. **Nadia Obi:** Writing – review & editing, Project administration. **Tilman Brand:** Writing – review & editing. **Hermann Brenner:** Writing – review & editing. **Laura Buschmann:** Writing – review & editing. **Renée T. Fortner:** Writing – review & editing. **Karin Halina Greiser:** Writing – review & editing. **Volker Harth:** Writing – review & editing. **Wolfgang Hoffmann:** Writing – review & editing. **André Karch:** Writing – review & editing. **Thomas Keil:** Writing – review & editing. **Alexander Kluttig:** Writing – review & editing. **Lilian Krist:** Writing – review & editing. **Michael Leitzmann:** Writing – review & editing. **Andy Maun:** Writing – review & editing. **Rafael Mikolajczyk:** Writing – review & editing. **Katharina Nimptsch:** Writing – review & editing. **Tobias Pischon:** Writing – review & editing. **Sabine Schipf:** Writing – review & editing. **Börge Schmidt:** Writing – review & editing. **Ulla T. Schultheiss:** Writing – review & editing. **Matthias Schulze:** Writing – review & editing. **Hajo Zeeb:** Writing – review & editing. **Christian Wiessner:** Writing – review & editing, Visualization, Software, Methodology, Formal analysis, Data curation.

## Declaration of competing interest

The authors declare that they have no known competing financial interests or personal relationships that could have appeared to influence the work reported in this paper.
